# Sustainability Indicators in Rice and Wheat Supply Chain

**DOI:** 10.3390/foods14162917

**Published:** 2025-08-21

**Authors:** Anulipt Chandan, Michele John

**Affiliations:** Sustainability Engineering Group, Curtin University, Perth, WA 6102, Australia

**Keywords:** sustainability indicators, rice supply chain sustainability, wheat supply chain sustainability, triple bottom line

## Abstract

Sustainability within the rice and wheat supply chain is integral to attaining the UN’s Sustainable Development Goals (SDGs), as they are the two most consumed grains as food. Rice and wheat cultivation significantly impacts the environment, with the agricultural sector employing 27% of the global workforce and contributing 4% to the world’s GDP, thereby affecting social and economic sustainability. Developing a sustainability index for the wheat and rice supply chain is a complex endeavor, as it depends on various factors such as the location of growers, farming methods, the target audience, and the stakeholders involved. This index must be derived from an optimal selection of indicators to avoid information overload while covering all essential sustainability aspects. There are different methods, such as life cycle assessment, energy analysis, ecological footprint, and carbon footprint, being used to assess sustainability, with indicator-based assessment emerging as a comprehensive approach. This study utilised the Triple Bottom Line (TBL) to identify optimal sustainability indicators in the wheat and rice supply chain. A systematic literature review was initially conducted, followed by an expert opinion survey to determine the required indicators. The literature review unveiled a wide array of indicators used across studies, often contingent on each study’s specific objectives. While some consistency existed in environmental indicators, discussions on social and economic dimensions within the wheat and rice supply chain were limited. Analysis of the expert opinion survey revealed a consensus on most selected indicators, albeit with variations based on experts’ geographical locations. The final set of optimal indicators identified can serve as a foundation for developing a sustainability index, implementing a sustainability information management system, and formulating policy initiatives in the rice and wheat supply chain.

## 1. Introduction

The concept of sustainability within the agricultural and food supply chain is both ambitious and ambiguous, as it involves a multitude of factors, each contributing to its assessment. It comprises different components, attributes, and indicators reflecting the complex interplay between the economic, social, and environmental aspects of sustainability, also known as the Triple Bottom Line (TBL) [1]. The sustainability concept proposed in the literature uses numerous indicators at various stages of the agricultural supply chain [2]. Sustainability indicators are increasingly seen as important tools in assessing agriculture supply chain sustainability, which varies according to time, location, stakeholder, and product [3]. However, existing development and critical analysis of indicators are not product-specific, leading to an incomparable sustainability index output and difficulties in sustainability data management due to a larger volume of data and a more complex supply chain [4]. Hence, sustainability indicator selection needs to explore product specifications as it provides key information on the supply chain’s environmental, economic, and social state.

Sustainable farming is important in achieving the UN SDG goal, facilitated by the integration of cutting-edge digital technology and information and communication technology (ICT). ICT plays a crucial role in enabling farmers to respond promptly to diverse challenges such as weather fluctuations, market assessments, sustainability monitoring, and enhancing supply chain efficiency [5]. It enhances economic sustainability by facilitating improved data management and analysis. Additionally, sensor technology and the Internet of Things (IoT) contribute to environmental sustainability by providing real-time data on farm management, promoting efficient resource utilisation. From a social sustainability standpoint, ICT supports initiatives like online education and telehealth services [5]. However, the integration of ICT faces challenges, including governance frameworks, human biases, uncertainties, and issues related to transparency and trust among supply chain partners. To overcome these challenges, recent studies advocate for the integration of blockchain technology, which enhances transparency in sustainability reporting and fosters consumer trust [6,7]. Nonetheless, the selection of appropriate indicators for blockchain-based sustainability reporting remains crucial. Therefore, this article evaluates a concise set of sustainability indicators necessary for the wheat and rice supply chain.

The primary aim of this paper was to establish a set of environmental, economic, and social indicators for evaluating the sustainability of the rice and wheat supply chain. This involved conducting a comprehensive systematic literature review and an expert opinion survey to identify an optimal set of indicators. The article is structured as follows: Section 2 describes the criteria for selecting indicators, Section 3 outlines the methodology employed, Section 4 provides a literature review, Section 5 details the expert opinion survey and its analysis, Section 6 discusses the limitations and implications, and finally, Section 7 concludes with observations.

## 2. Indicator Selection Criteria

Sustainability assessment is a complex process and requires the consideration of numerous variables. Central to this assessment is the careful selection of sustainability indicators, as they significantly influence the outcome of indicator-based sustainability assessments [8]. Numerous indicators are available in the literature for evaluating wheat and rice supply chain sustainability. Nevertheless, an excessive number of indicators can complicate data collection, validation, and analysis, while too few may overlook critical aspects of sustainability. Consequently, identifying the optimal set of indicators poses a significant and challenging task. Reliable and credible sustainability information depends on well-defined and optimally selected indicators [9]. Therefore, a robust procedure is imperative to choose and define indicators, offering conclusive evidence on sustainability to inform future policy design and improvement efforts. This paper employs a three-step process for selecting sustainability indicators: the contextualisation and comparison of indicators, and final selection [9].

### 2.1. Contextualisation

The initial step in indicator selection involves contextualisation, which entails making preliminary choices and assumptions for sustainability analysis. This encompasses defining the sustainability concept, outlining study objectives, and identifying relevant stakeholders [8]. The selection process involves defining the context and perception under which indicators were chosen, as their significance varies based on the context and stakeholders. Moreover, specifying the assessment’s end users is crucial, as the chosen indicators may differ depending on whether they are researchers, farmers, decision-makers, or consumers [8].

The contextualisation of this study aimed to identify the optimal set of sustainability indicators within the wheat and rice supply chain. Utilising the TBL framework, indicators were categorised under environmental, economic, and social dimensions. The selection process involved considering all stakeholders in the supply chain, with a focus on consumers as users of the sustainability index. Balancing the need for comprehensive information with the risk of overload, this study sought to determine an appropriate number of indicators that offer sufficient sustainability insights for informed decision-making [8].

### 2.2. Comparison and Evaluation of Indicators

Selection criteria were established to systematically compare and evaluate indicators found in the literature, ensuring transparency in the selection process. These criteria vary based on the context and objectives of the sustainability assessment [10].

In conducting the assessment, chosen indicators should align with the specific objectives, the system under consideration, and the scales of analysis. Sustainability assessments for rice and wheat supply chains encompass various spatial scales, including farm-level, pre-and post-production, regional, and national levels [11].

Dynamic scale indicators are essential for monitoring the grain supply chain’s status at various points or comparing it with the reference values [12]. The dynamic nature of agricultural systems necessitates frequent measurements to capture fluctuations accurately. These dynamic indicators should be measured at intervals (e.g., annually, medium term, long term) that highlight significant variations and the impact of specific parameters.

Conversely, static-scale indicators should account for variability stemming from external factors. For instance, income may exhibit high variability within a single year, prompting calculation based on a 3-year average [12]. Therefore, classifying indicators based on data collection methods is crucial for effective comparison and analysis.

### 2.3. Selection

Ensuring the suitability of indicators according to stakeholders’ expectations is crucial in sustainability assessments. Indicators should be clear, readable, and easily understandable for target users. They serve as a means to convey meaningful information while simplifying intricate processes.

There are two main factors for the selection of indicators: practical value and appropriateness [2,8]. Practicality is paramount, encompassing data availability, measurability, and calculation approaches. Indicators should be calculated from easily available and obtainable data, i.e., existing databases, directly obtained from farms or collected at a reasonable cost and within a reasonable time. Data availability is the first filter applied in the selection process in data-driven approaches [13].

Once the suite of indicators is finalised, the next step involves integrating them into a unified index that can be comprehended and reported independently. This integration process entails assigning weights and aggregating the various components of the index [4]. These weights are typically determined based on statistical criteria or expert judgment. The sustainability index, formed by aggregating sustainability indicators, is crucial for effective communication. However, the relevance of aggregation is often debated due to potential information loss, methodological challenges, subjective weighting of components, and defining reference values [14].

## 3. Methodology

**Figure 1:**
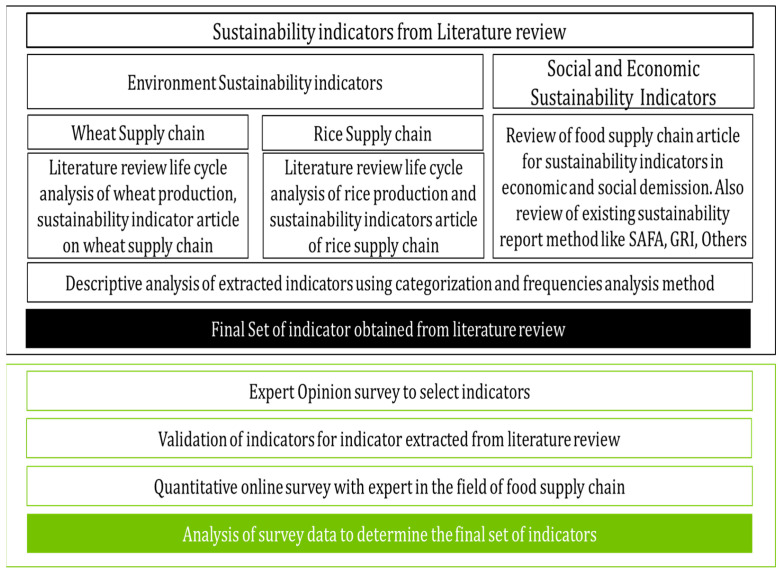
Research methodology.

This study employed a two-step approach to identify the optimal sustainability indicators within the rice and wheat supply chain, as illustrated in Figure 1. Firstly, a systematic literature review was conducted to extract key indicators under the TBL framework. Subsequently, an expert opinion survey was conducted to refine the extensive pool of indicators, aiming to deliver a concise set of sustainability indicators.

### 3.1. Systematic Literature Review

In the initial step of this study, a systematic literature review of the rice and wheat supply chain was conducted following a four-step model to ensure the selection of indicators’ reliability and robustness. This model encompassed material collection, descriptive analysis, categorisation, and evaluation.

The systematic literature review process began by defining and limiting the selection of material for subsequent analysis. Aligned with the research objectives, a structured keyword search was conducted across four key scientific databases: Scopus, Wiley, IEEE Xplore, MDPI, and Web of Science. Keywords such as “sustainability indicators in wheat supply chain”, “sustainability indicator in rice supply chain”, “sustainability indicators in food supply chain”, “LCA analysis in wheat”, and “LCA analysis in Rice” were utilised.

Initially, a scientific database search yielded 269 articles, which was subsequently refined by eliminating duplicates and non-English articles, resulting in 109 papers for further analysis. Subsequently, articles unrelated to the wheat and rice supply chain for environmental indicators and those unrelated to the food supply chain for economic and social sustainability indicators were excluded. Only articles published in peer-reviewed journals and reputable conferences were considered to ensure the paper’s reliability and quality. After this filtration process, 77 articles remained for a final review to extract sustainability indicators under the Triple Bottom Line (TBL).

The chosen articles were categorised according to the wheat and rice supply chain, with environmental indicators segmented into rice and wheat categories, while economic and social sustainability indicators were grouped together for both grains. This classification was based on the understanding that only production and processing methods vary between different food products, impacting environmental sustainability, while economic and social sustainability aspects remain largely consistent across the food supply chain.

After categorisation, the articles were split into two groups. The first category comprised 44 articles focusing solely on examining the environmental sustainability aspects of wheat and rice production. Meanwhile, 33 articles were reviewed to extract insights into economic and social sustainability within the food supply chain. This was prompted by the limited analysis of economic and social sustainability in the wheat and rice supply chain.

### 3.2. Expert Opinion Survey

In the second step of this study, an online survey was administered to stakeholders across the grain supply chain, focusing on both wheat and rice sectors. The survey encompassed a range of environmental indicators specific to the wheat and rice supply chains, along with commonly used social and economic sustainability indicators. Experts from the wheat and rice farming sectors were surveyed to evaluate the impact, effectiveness, and significance of an initial set of indicators.

## 4. SLR on Rice and Wheat Supply Chain Sustainability Indicators

The environmental footprint of rice and wheat production is notable, influenced by factors such as fertiliser and pesticide usage, irrigation water consumption, and energy used by farm machinery. Agricultural practices exhibit regional variations, thereby affecting environmental impacts differently across regions. Generally, indicators fall into four categories: means-based indicators, system–state indicators, emission indicators, and effect-based indicators. Means-based indicators evaluate the technical inputs employed in farming, while system–state indicators gauge the overall condition of the farming system. Emission indicators focus on the pollutants emitted by farms and their potential environmental consequences, while effect-based indicators measure the direct impact of farming practices on the environment [15]. This study adopted a means-based approach to identify indicators aligning with the TBL framework.

### 4.1. Environmental Sustainability

#### 4.1.1. Wheat

Wheat holds a vital position as a staple food for human consumption, finding its way into various food items such as bread, pasta, noodles, pastries, rolls, and biscuits. Cultivated across diverse environmental settings worldwide, wheat stands as the most consumed grain as food, ranking second in global production [16].

Various methodologies are being employed in the literature to evaluate the impact of wheat production, including energy analysis, carbon footprint analysis, water footprint analysis, input–output energy analysis, and Life Cycle Assessment (LCA) [4]. Of these, LCA emerges as the preferred method for assessing environmental impact. LCA studies exhibit diversity in their objectives, system boundaries, impact categories, and inventory. Nonetheless, there are common elements among the studies, such as climate change being a prevalent impact category, while inputs such as fertilisers, water, and machinery are consistently considered input across the reviewed studies.

Pesticides and fertilisers stand as key inputs in wheat production. Pesticides serve to mitigate plant diseases and enhance productivity. Meanwhile, fertilisers play a pivotal role in raising yield, with potential increases of up to 50%, contingent upon the dosage applied [17]. However, their application exerts adverse effects on freshwater resources and exacerbates climate change, chiefly due to substantial greenhouse gas (GHG) emissions [18,19,20]. In [21], a comprehensive meta-analysis of LCA studies conducted within the EU region highlights that both the production and application of fertilisers are responsible for up to 83% of the total Global Warming Potential (GWP). Notably, fertiliser production emerges as the primary contributor to GHG emissions, while field emissions stemming from the runoff of applied mineral fertilisers significantly impact marine ecotoxicity [22].

Capital goods such as machinery and buildings significantly affect environmental sustainability. Machinery utilised in farming activities and the construction of buildings collectively contribute more than 10% to total GHG emissions [23]. A sensitivity analysis of system boundaries has revealed that machinery production notably impacts all environmental impact categories, particularly human toxicity (HT) and marine ecotoxicity, with a magnitude exceeding 50% [22]. Conversely, the construction of buildings significantly influences climate change. The GHG contribution of soil organic carbon (SOC) stock changes amounting to 200 kg CO_2_ eq per hectare. This contribution is substantial compared to N_2_O emissions [24].

Fertilisers and machinery serve as significant sources of greenhouse gas emissions widely employed in medium and large-scale agricultural operations. Thus, comprehending the influence of farm size on environmental performance becomes imperative. Studies examining farm size as a variable indicate that larger farms tend to exhibit lower emissions compared to smaller farms [25,26,27]. The definition of a large-sized farm varies across studies. For instance, in [27], farms encompassing an area greater than 6.7 hectares, classified as large farms, demonstrated a 17% reduction in carbon footprint per unit area compared to small-scale farms. Similarly, in another study, the amalgamation of fragmented land to form larger farms resulted in a reduction in environmental impact indices based on both area and yield by 28.8% and 18.3%, respectively [28]. This reduction can be attributed to enhanced farm management practices observed in larger farms, facilitating the optimal utilisation of machinery, fertilisers, and irrigation systems.

The environmental performance of wheat production is heavily influenced by on-farm management practices. These practices encompass both on-farm and pre-farm activities, which dictate inputs such as the type and quantity of fertiliser, use of pesticide, use of farm machinery, tillage and stubble management, and practice of intercropping. These factors collectively impact emissions from the farm [18,20,26]. For instance, employing a legume intercropping practice in wheat production has been shown to reduce GHG emissions by 56% per hectare and 35% per ton yield basis [29]. Additionally, in scenarios where no fertilisers are used, GHG emissions were lower compared to scenarios involving fertiliser application [30]. This underscores the significant influence of farm management practices on environmental sustainability.

The utilisation of irrigation systems has significant environmental impacts, notably on freshwater resource depletion and GHG emissions attributed to electric energy usage [17,31]. The machinery employed for irrigation operations necessitates substantial amounts of electricity to extract water from aquifers, thereby contributing to GHG emissions. Typically, irrigation water is sourced from shallow aquifers, surface water bodies, and deep aquifers. As the depth of the water source increases, the energy requirement for pumping water also increases, consequently leading to higher emissions. Moreover, the extraction of water from deep aquifers can result in a decline in groundwater levels. It is worth noting that irrigation activities can account for up to 10% of the total GHG emissions associated with wheat production [27].

This review highlights the multitude of variables influencing the environmental performance of wheat production. It indicates that the primary factor affecting environmental sustainability is the application and production of fertilisers. Additionally, the use of pesticides and fossil fuels in machinery plays a significant role in impacting environmental sustainability. The selection of function units and system boundaries in LCA studies also influences the sustainability outcomes. Moreover, the consideration of impact categories in LCA studies emerged as another crucial factor that shapes the perception of sustainability.

#### 4.1.2. Rice

Rice stands as a significant agricultural commodity globally, serving as a staple food in Asia, Latin America, and Africa. The expected population growth in major rice-consuming regions indicates a projected increase in both rice production and consumption [16]. While rice production contributes to rural livelihoods by providing income, food, and feed, it also poses significant environmental challenges. The environmental footprint of rice production is substantial, primarily due to methane emissions from flooded fields, fertiliser usage, and high freshwater and pesticide consumption [32]. Therefore, understanding the myriad factors influencing the environmental performance of rice production is crucial for envisioning sustainable rice cultivation practices.

GHG emissions emerged as the predominant environmental impact analysed across all reviewed articles pertaining to rice production. Alongside Global Warming Potential (GWP), freshwater usage was identified as another crucial environmental factor. However, it’s worth noting that water sourced for irrigation from channels or similar non-groundwater sources, which necessitates no electricity and has no impact on groundwater, does not contribute to environmental degradation [33].

Rice cultivation is characterised by its high demand for water, as fields are often flooded for extended periods during the growing season. This practice entails significant freshwater usage, which not only affects groundwater levels and other water bodies, but also contributes to water table depletion. Water consumption in rice farming ranks as the second most prominent environmental impact following methane emissions. The inundation of rice fields necessitates substantial electricity usage, leading to additional greenhouse gas emissions. According to [34], electricity consumption for irrigation purposes constitutes approximately 6–11% of total energy usage. In contrast, another study [35] indicates that irrigation-related activities, including fuel usage and machinery production, contribute to 53.65% of GWP. Rice cultivation occurs across different seasons, resulting in varying irrigation requirements. During the dry season, irrigation needs are heightened compared to the wet season, leading to increased energy, labor, and water demands and consequently higher emissions [11]. Research depicted in [33] revealed that rice production of identical varieties during summer and spring seasons yielded equivalent methane emissions, despite a 4% increase in irrigation energy requirements during summer crops compared to spring. In conventional rice farming practices, fields are flooded throughout the cultivation period, creating anaerobic conditions conducive to methane emissions, which constitute a significant portion of greenhouse gas emissions. According to [36], methane emissions from fields account for 76.85% of the total greenhouse gas emissions from unmilled rice. Moreover, findings from [37] indicate that the cultivation stage contributes to 95% of the total GWP in the rice production system.

Unlike conventional rice farming practices, the System of Rice Intensification (SRI) method adopts intermittent flooding, leading to a reduction in greenhouse gas (GHG) emissions. The research presented in [38] conducted on the SRI farming approach demonstrated a 60% decrease in GHG emissions compared to conventional methods, attributable to diminished field emissions and increased yield. Additionally, water consumption was slashed by 2.5 times per kilogram of yield, resulting in reduced energy requirements for irrigation and subsequently lower emissions. Moreover, the SRI method entails substantially lower usage of fertilisers and pesticides compared to conventional practices.

The production and application of synthetic fertilisers in rice farming represents another significant contributor to GHG emissions. According to [33], fertilisers’ production and application contribute to 19% of total emissions. The optimal rate of fertiliser application varies with production, reaching a point of maximum efficiency. However, surpassing this optimal point leads to a higher environmental impact per unit [39].

Farm management practices exhibit variability from one farm to another, leading to differences in greenhouse gas emissions. Various tillage methods, such as no-tillage and minimum tillage, are employed in rice farming. In instances of organic farming, where synthetic fertilisers are eschewed, greenhouse gas emissions tend to be lower compared to conventional farming methods on a per-area basis [40]. Similarly, the findings in [41] suggest that upland and organic farming demonstrate superior environmental performance in terms of area coverage. However, contrasting viewpoints are presented in [42], arguing that organic farming may not be environmentally friendly due to higher GWP, water depletion, and acidification potential.

The environmental footprint of rice production is predominantly influenced by methane emissions, fertiliser application, fossil fuel usage, water consumption, and electricity usage. Additionally, the selection of system boundaries and variables considered in the analysis significantly affects environmental performance [32,36]. Hence, establishing a consistent and well-defined system boundary is crucial for obtaining a comprehensive environmental sustainability indicator for rice production.

### 4.2. Economic Sustainability

Economic sustainability refers to the financial feasibility of farming systems, indicating their capacity to generate profits and ensure prosperity within the farming community. This aspect is closely intertwined with the social dimension, as income levels play a critical role in facilitating access to various social activities.

Ensuring economic viability is crucial for the sustainable development of farms and businesses. The production stage of agricultural produce significantly influences the variability of environmental sustainability. However, the factors affecting the economic sustainability of wheat and rice production are similar to those impacting other agricultural products. Thus, examining economic sustainability indicators in agriculture offers insights into key economic considerations.

Various indicators were employed in the article to gauge the economic performance of agricultural products. The review revealed a diverse array of economic indicators in the literature, albeit with a focus on some common metrics such as profitability and farm income. Productivity or yield emerged as the most frequently utilised economic indicator, calculated in different forms such as land productivity, labor productivity, and input productivity [14]. Land productivity, measured as produce per unit area, serves as an economic benchmark for the product, although its economic value can vary depending on the market. Another significant indicator examined in the study was farm profitability, directly reflecting the economic sustainability and resilience of the farm [43]. While profitability stood out as one of the most prevalent economic indicators across studies, it was often referred to by different names with similar objectives [44]. Various indicators akin to profitability were utilised in the studies, including the benefit-to-cost ratio [14] and input productivity, representing the value of production per unit input cost. Labor productivity, another productivity indicator explored in the study, denotes the output produced per unit of labor employed.

Farm revenue stands as another significant economic indicator examined in studies. With objectives akin to determining farm revenue, indicators such as net farm return, farm income, revenue management, and agricultural producers’ income were considered. Production cost emerged as another indicator assessed in some studies, with the aim of enhancing the economic performance of a product by reducing costs. Cost optimisation, as highlighted in [27], was regarded as a crucial economic concern. The review underscores the interconnectedness of profitability, production cost, and farm revenue. While these indicators share similar objectives, profitability holds greater relevance as it encompasses both revenue and production costs.

Another crucial aspect of economic indicators pertains to the efficient utilisation of labor [44]. Labor productivity, an economic metric, signifies the output produced per unit of labor employed. Increased labor productivity leads to a reduction in total production costs, thereby correlating with improved economic sustainability [44]. In the context of family farms where all family members are engaged in farming activities, assessing productivity poses challenges due to the time devoted to farm work. Consequently, [13] considers income per family working member as an economic indicator. A review underscores that optimising labor utilisation enhances economic performance. Hence, the number of laborers employed per unit serves as an indicator of a farm’s economic vitality.

Farmers’ earnings play a pivotal role in determining the economic sustainability of a product. Various indicators such as Potential Income of the Farmer, Available Income per Worker compared with the national legal minimum wage, and farmers’ income were utilised in a study to depict farmers’ earnings. Farmer income is intricately linked with the economic viability of the farm. In [43], investment viability was incorporated as an economic indicator to assess whether a farm is economically feasible. A significant portion of farmer income stems from the sale of products, comprising both direct and indirect income from agricultural tourism. Additionally, farmer income is influenced by subsidies they receive, making subsidies an important economic indicator featured in several studies [13,43].

In essence, economic indicators serve as quantitative measures of a farm’s financial viability, presented either in monetary terms or as ratios. The primary economic indicators typically revolve around the profitability of a farm, encompassing factors such as farm income, efficiency, and productivity. These indicators can be classified into three main objectives: (1) management of the farming system concerning external inputs like feed concentrates or mineral fertilisers, subsidies, and external financing; (2) income diversification, achieved through production activities, non-production endeavors like agritourism, and marketing; and (3) the long-term sustainability of the farm, particularly concerning succession and transmission. Indicators aligned with these objectives assess the farm’s ability to adapt to changes in the external context, such as fluctuations in the prices of agricultural products and inputs like energy.

### 4.3. Social Sustainability

Quantifying social sustainability poses challenges due to the subjective differences among supply chain stakeholders’ perspectives and the limited availability of necessary data [45]. Social sustainability is defined at both the farm community level, capturing internal social objectives concerning the well-being of the farmer and their family, and at the societal level, encompassing external social objectives shaped by society’s evolving values and concerns. As a result, the definition of the social sustainability dimension remains in a state of flux, reflecting the dynamic nature of societal expectations and priorities [46].

In contrast to environmental and economic sustainability, social sustainability receives less attention within the TBL framework, despite its equal importance in fostering sustainability in the agriculture sector [14], measuring social sustainability poses significant challenges due to its vague and subjective definition [45]. Nonetheless, various indicators have been proposed in the literature to quantify social sustainability and capture the social value embedded within a food supply chain. External and internal supply chain drivers and strategies play crucial roles in sustainability information [47]. Therefore, a curated selection of indicators is necessary to effectively gauge sustainability performance. Various frameworks have been suggested in the literature for selecting social sustainability indicators. In [46], a framework was introduced to evaluate social sustainability within the food supply chain. This framework incorporated a feedback loop aimed at enhancing sustainability performance. Such frameworks illustrate that sustainability performance is dynamic and necessitates a tailored set of context-specific indicators.

In [46], indicators were classified into five key categories: biodiversity, nutrition, information and communication, resource use and pollution, and value creation and distribution. The analysis revealed overlapping indicators across the TBL, making it challenging to categorise indicators under individual TBL dimensions. The literature review identified numerous social indicators utilised in food supply chains. Among the most commonly employed social indicators were education, knowledge, employment, average wages, equality, and accident rates. Additionally, in [48], a matrix was introduced to assess social sustainability indicators, encompassing factors such as average wages per person, freedom of employment, community engagement, and product and service failure. In [44], the availability of social sustainability indicators is constrained by data limitations. The chosen social sustainability indicators are categorised into three themes: employment volumes, quality of employment, and gender balance, each theme featuring one indicator. The selected indicators include average wages, number of employees, and the female-to-male ratio.

Workplace safety incidents are emerging as key indicators in the food supply chain, attributed to the utilisation of heavy machinery, pesticides, and fertilisers, which pose health risks to workers [49]. Furthermore, the health and safety of workers are pivotal sustainability metrics. Working conditions encompass factors like working hours and contractual agreements, while health and safety considerations involve occupational health, safety protocols, and workplace environments. Various indicators are employed in the literature to assess workplace working conditions [2].

Another significant group of sustainability indicators pertains to the knowledge and training of workers. Providing farmers with training in new techniques, such as disease management, enhances competitiveness. As highlighted in [10], targeted training is recognised as a crucial indicator reflecting the social well-being of employees. Social capital and community engagement are two additional indicators that researchers have identified as important metrics for assessing social sustainability [14].

A list of indicators obtained from the literature review is presented in Appendix A.

## 5. Development of SI in Rice and Wheat Supply Chain: An Expert Survey

### 5.1. Participants

Using the indicators derived from the SLR, a preliminary set of sustainability indicators was chosen for inclusion in the survey. The survey was disseminated among experts through an online survey platform to gather their opinions on the significance of these indicators. Experts were invited from diverse agricultural sectors, including farming, marketing, processing, policymaking, and academia.

### 5.2. Survey Design

The number of participants in expert opinion surveys has shown variability across studies, influenced by factors such as the research objectives, participant selection criteria, methodological approach, and validation procedures. Reported participant sizes have ranged from 20 to 60 individuals [14]. In this study, a total of 50 respondents’ opinions were selected for final analysis. Restricting the size of participants offers advantages such as efficient communication with experts, and streamlining the data filtering and analysis processes. Participants were carefully screened based on their expertise and years of experience in the agricultural and sustainability sectors. Screening criteria were established, requiring a minimum of 15 years of involvement in the grain supply chain and sustainability field for inclusion in the survey.

### 5.3. Survey Distribution and Data Collection

An online survey tool (Qualtrics.com) was employed to administer the survey. The survey questionnaire was thoroughly drafted based on the indicators extracted from the literature review of the rice and wheat supply chain, utilising frequency analysis to identify relevant indicators. Only indicators appearing in three or more articles were considered for inclusion. Consequently, the rice supply chain featured 15 environmental indicators, while the wheat supply chain comprised 14. For economic sustainability, 12 unique indicators were selected, whereas the social dimension yielded 10 due to limited findings.

In order to gather more thorough feedback from experts and enhance the reliability of the survey, an option was incorporated where experts could propose additional indicators; they were deemed significant but were not initially included in the survey. Each indicator was assessed using a Likert scale spanning 1 to 5, with 1 representing high importance and 5 indicating irrelevance. Indicators were organised according to the TBL, with environmental factors further divided into rice and wheat categories. Additionally, in the survey, each indicator was accompanied by a concise definition and measurement unit to elucidate its intended meaning and application.

To enhance the validity of the survey questionnaire, the initial draft was subjected to consultation and validation by two industry experts. Following their feedback, adjustments were made, including the incorporation of additional indicators suggested by the experts. For example, one of the suggested indicators was the use of lime on farms to mitigate soil acidity, a practice commonly employed in Australian farming contexts but not prevalent in Asian farming literature. Despite this discrepancy, the suggested indicators were included in the final draft of questions for the online survey platform, ensuring comprehensive coverage of relevant sustainability indicators across different agricultural contexts.

The survey link was shared through email and social media platforms, employing a personalised approach. Initially, experts were contacted via email, where they were provided with an information sheet outlining the survey’s purpose and applicable ethical approvals. Additionally, some industry experts were reached out to via social media channels. Given the screening criteria embedded within the online survey, the likelihood of receiving invalid responses was minimal. Moreover, the data obtained from the screening criteria were utilised to categorise experts based on their experience level and respective employment sectors.

Out of the 80 experts contacted for the survey, only 59 participants responded and completed the survey. However, only 50 responses were selected for further analysis after validation and filtering. The collected survey data from the online platform were then extracted into an Excel sheet for analysis.

### 5.4. Demographic Analysis of Survey Participants

**Figure 2:**
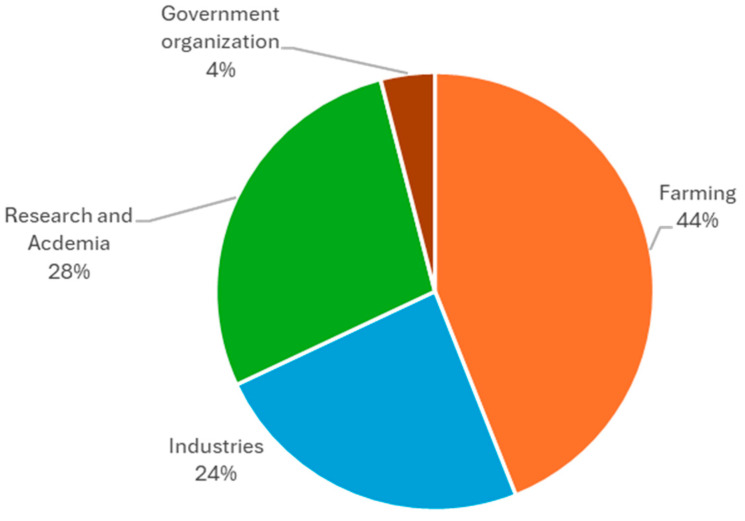
Industry-wide distribution of survey participants.

Among the survey respondents, 44% hailed from the farming sector, 24% represented industries, 28% were associated with research and academia, and 4% were affiliated with government organisations. Noteworthy participants in the farming sector encompassed grain farmers, farm managers, and proprietors. Meanwhile, individuals from the industry sector comprised a diverse range of roles, including managing directors, business owners, managers, senior managers, and agronomists. Participants from the research sector included agriculture scientists, researchers, and professors, indicating a broad spectrum of expertise and professionalism across different fields (Figure 2).

The distribution of participant experience in agricultural domains, particularly the substantial proportion reporting 10–20 years of experience (56%), highlights the wealth of practical knowledge and industry insights available to inform sustainability advancements. Moreover, the significant percentage of respondents with 21–30 years of experience (30%) indicates a cohort of seasoned professionals who have witnessed and contributed to the evolution of sustainability practices over several decades. 

Demographic data from the survey reflect geographical diversity: (52%) were from Australia, while 20% hailed from the US, 12% from India, and 16% from the UK. Geographical diversity among survey respondents further enhances the study’s credibility and applicability by capturing perspectives from various global contexts. The predominant representation from Australia, alongside participants from the US, India, and the UK, ensures that a broad spectrum of environmental conditions, regulatory frameworks, and cultural contexts are considered.

The comprehensive representation of stakeholders from the farming, industry, research, and academic sectors, coupled with varied levels of experience and global perspectives, enhances the robustness and relevance of the study’s findings.

### 5.5. Analysis

The survey data were analysed using SPSS statistical software version 28. Initially, a data cleaning process was performed, involving the screening of data to identify and remove any erroneous entries. After eliminating false data, the cleaned dataset was imported into the SPSS software for further statistical analysis.

#### 5.5.1. Rice Supply Chain Environment Indicator

The survey analysis on environmental indicators in the rice supply chain provides valuable insights into expert perceptions regarding sustainability metrics, highlighting both consensus and divergence among experts. Table 1 presents the analysis of expert opinions regarding environmental indicators in the rice supply chain.

Fertiliser use stands out as a critical environmental indicator, with the lowest mean value of 1.72 and the lowest standard deviation of 0.834. This indicates a consensus among most experts regarding the high importance of fertiliser use in the rice supply chain. The significant impact of fertiliser on environmental factors such as GHG and eutrophication underscores the need for balanced fertilisation practices to mitigate environmental degradation while maximising productivity.

Conversely, fertiliser produced on-farm was ranked as least important with a mean value of 2.6, reflecting the practical limitations and prevalence of off-farm fertiliser sources. This finding highlights the importance of considering practical feasibility and regional agricultural practices in sustainability assessments.

Weed management strategies, such as integrated weed management with a mean value of 1.92 and pesticide usage mean value of 2.12, were identified as crucial environmental indicators, reflecting their impact on soil health and ecosystem sustainability in rice farming.

The importance of co-product and waste management, as well as packaging material mean value of 2.18, was recognised by experts, despite receiving less attention in the literature. This highlights the need for comprehensive sustainability assessments that encompass diverse environmental impact factors beyond traditional metrics.

Fuel usage, and its associated GHG emissions from farm machinery and aerial applications were acknowledged as critical environmental factors, emphasising the need for improved fuel efficiency and alternative energy sources in agricultural operations.

Field emissions, particularly methane gas generated from flooded rice fields, were identified as a significant environmental concern with a mean value of 2.22, corroborated by both the survey results and literature review findings. The high mean value assigned to field emissions underscores its importance in rice production sustainability assessments.

Lime usage, despite being mentioned less frequently in the literature, was deemed important by experts, indicating its regional significance in soil management practices. This finding underscores the importance of considering localised agricultural practices in sustainability assessments.

Overall, the survey analysis provides valuable insights into the complex relationship of environmental factors in rice production sustainability, emphasising the importance of considering regional variations, practical feasibility, and holistic environmental impact assessments of rice supply chain in sustainability evaluations.

#### 5.5.2. Wheat Supply Chain Environment Indicator

The analysis of environmental indicators in the wheat supply chain reveals insights into expert perceptions and the relative importance assigned to different sustainability metrics presented in Table 2.

The survey analysis highlights variability in expert opinions, with some indicators being deemed highly important like water use while others are considered less significant like fertiliser produced at farm. The analysis also revealed that the ranking of indicators differs between rice and wheat. This disparity underscores the nuanced nature of environmental sustainability assessment and the need for comprehensive evaluation frameworks.

Lime usage emerges as a critical indicator, with a mean value of 2.16, particularly in acidic soil regions like North America and Australia. However, its importance varies across regions, as evidenced by its omission in the literature review focused on Asian countries. The inclusion of lime usage in the survey underscores the relevance of localised agricultural practices and the influence of regional factors on sustainability assessments.

Conversely, fertiliser produced on-farm was ranked the lowest, with a mean value of 2.62 as the least important indicator, aligning with findings from the literature review. Pesticide usage emerges as a significant indicator, with a mean value of 2.12, reflecting its widespread use in farming practices and its environmental implications. The alignment between survey results and literature findings underscores the importance of pesticide management in sustainable agriculture.

The variability in expert opinions is further illustrated by the standard deviation and variance of indicators such as machinery used on-farm, indicating divergent views among experts. This divergence underscores the complexity of sustainability assessments and the multifaceted nature of environmental impact considerations.

Despite its limited mention in the literature, the cropping program indicator’s importance underscores its relevance in enhancing productivity and reducing resource use. This finding highlights the need for comprehensive sustainability assessments that consider diverse factors beyond traditional metrics.

Similarly, despite receiving less attention in the literature, co-product and waste management handling emerges as crucial indicators, having a mean value of 2.24 in expert opinions. This discrepancy underscores the importance of integrating diverse perspectives and considering holistic approaches to sustainability assessment.

Overall, the analysis underscores the complexity of environmental sustainability assessment in the wheat supply chain, and the importance of integrating diverse perspectives, regional variations, and practical realities into evaluation frameworks.

#### 5.5.3. Economic Indicator

Economic indicators play a pivotal role in assessing the financial health and sustainability of the rice and wheat supply chain. In this study, economic indicators were adapted from the Food Supply Chain (FSC) due to the scarcity of the literature specifically addressing economic aspects within the rice and wheat supply chain sustainability domain. Despite the vast array of economic indicators identified in the literature review (89 distinct indicators), only a handful (11 indicators) were recurrently mentioned in multiple articles, indicating a lack of consensus or standardisation (Table 3).

The majority of economic indicators included in the survey were deemed significant, as they were derived from the literature review findings and subsequently validated through survey responses. Interestingly, there was minimal variance in the mean values (1.86 to 2.32) of most indicators across the survey analysis. This uniformity in mean values suggests a general consensus among experts regarding the importance of these economic indicators.

The survey results highlighted revenue as the foremost economic factor with a mean value of 1.86, whereas experts perceived production cost with a mean value 2.32 as relatively less significant. Moreover, the benefit-to-cost ratio emerged as a crucial economic indicator, characterised by low standard deviation (0.903), signifying a high level of agreement among experts regarding its importance. Conversely, profitability elicited more diverse opinions among experts, as evidenced by its higher standard deviation value (1.273).

Revenue, which emerged as the most significant economic sustainability indicator with a mean value 1.86, is essential for a farm’s financial health and its ability to diversify income sources. Generating higher revenue translates to increased income for both farmers and farm workers, contributing to the overall economic stability within the agricultural sector.

Land productivity emerged as the second most critical economic sustainability indicator, with a mean value 1.94 based on both survey analysis and literature review findings. Land productivity is a critical economic indicator that measures the amount of grain produced per unit of available land. It plays a crucial role in determining the economic viability of a farm, as higher land productivity leads to increased crop production and, consequently, higher income levels. Previous research has underscored the importance of land productivity in assessing economic sustainability within the FSC, emphasising its dependency on factors such as soil fertility, environmental conditions, and technological advancements [14]. This alignment underscores the pivotal role of land productivity in determining agricultural profitability and efficiency. The prevalence of land productivity as a key economic indicator in the literature further validates its significance in assessing economic sustainability within the rice and wheat supply chain context.

The production cost of rice, which varies based on factors such as rice type and energy consumption during production processes, is a crucial economic consideration. However, the benefit-to-cost ratio, despite its high mean value, was infrequently utilised in the literature, potentially due to the prevalence of other indicators like productivity and revenue in economic sustainability evaluations.

Profitability, indicating the economic returns on investments in the FSC, received higher mean values of 2.18 compared to other surveyed indicators. While its significance may not be adequately reflected in mean values alone, the higher standard deviation (1.273) and variance suggest varied expert opinions on its importance, possibly influenced by the prioritisation of other indicators such as productivity and benefit-to-cost ratio.

Production costs encompass various expenses such as labor, raw materials, and energy, providing insights into the total input costs of agricultural operations. However, its utility in assessing the economic viability of the FSC may be limited, as evidenced by its highest mean value 2.32 in survey analysis and its infrequent use as a social sustainability indicator in literature.

Overall, while land productivity and revenue emerged as pivotal economic indicators, variations in the importance attributed to profitability and production costs highlight the complexity of economic sustainability assessment within the rice and wheat supply chain context. Further research and refinement of economic indicators are warranted to enhance the comprehensiveness and applicability of economic sustainability evaluations in agricultural settings.

#### 5.5.4. Social Indicators

Social indicators, similar to economic indicators, were deduced from the FSC literature review due to the limited literature addressing social sustainability in the wheat and rice supply chain. Although the social aspect remains the least explored dimension under the TBL in the literature, its significance in the rice and wheat supply chain cannot be understated, especially considering the substantial workforce involved, particularly in developing nations. The survey analysis underscores the high importance attributed to all social indicators, with mean values ranging from 2.00 to 2.60 (Table 4).

The analysis provides a comprehensive overview of key social indicators in the context of agricultural sustainability, shedding light on their significance and expert perceptions. The identification of the accident rate as a pivotal social factor, supported by its lowest mean value (2.00) with standard deviation of 1.088, showed that the experts’ responses were relatively consistent and alignment with literature findings, underscores its critical role in ensuring worker’s safety. The standard deviation of 1.088 suggests that while there was some variation in the responses, it was not substantial. This consensus highlights the universal recognition of the accident rate’s importance in safeguarding agricultural workers.

The observation that education level received comparatively lower importance with a mean value of 2.3 in the survey, despite its universal significance, raises questions about the nuanced distinction between formal education and job-specific training. This discrepancy emphasises the need for a more nuanced understanding of the role of education versus training in promoting social sustainability.

The high importance attributed to community engagement, with a mean value of 2.26 in both the survey and literature review, underscores its crucial role in fostering sustainable social development. This consensus reflects the universal acknowledgment of community engagement as a key driver of social sustainability, aligning actions with community needs.

The scattered expert opinion on gender equality with a high standard deviation of 1.22 highlights the complexity of addressing gender dynamics in agriculture, particularly regarding women’s involvement in farming versus other supply chain activities. This divergence underscores the need for tailored interventions to address gender disparities effectively.

The unanimous recognition of training and employment with the same mean value of 2.14 as a crucial indicator emphasises its role in enhancing employees’ knowledge and efficiency, reflecting a consensus among experts on its significance for social sustainability. The identification of employment as a cornerstone of social sustainability echoes its pivotal role in generating livelihood opportunities, emphasising its importance across rural and urban areas.

The acknowledgment of fair wages, with a mean value of 2.18 as essential for supporting productive employment and social development, underscores their significance in promoting social equity and economic stability.

The relatively neutral importance level with a mean value of 2.2 attributed to health practices in the survey suggests a potential oversight or undervaluation of health-related indicators. This discrepancy underscores the need for a more comprehensive approach to addressing health practices within the context of social sustainability, considering their multifaceted impact on worker well-being and productivity.

## 6. Discussion

This study employed a two-step approach to select sustainability indicators within the wheat and rice supply chains using the TBL framework. In capitalist markets, sustainability is often seen as achievable only when it aligns with the economic bottom line, ensuring companies’ continued operation. While sustainability may not be the primary focus of a profit-driven supply chain, some leading industries prioritise sustainability in their branding and overall strategy. This strategic approach enhances their reputation and demonstrates their commitment to environmental stewardship to stakeholders.

Amid increasing consumer demand for sustainable food products, food companies are compelled to take proactive steps toward sustainability. Despite the widespread acknowledgment of the importance of sustainability, there remains a lack of clear, quantitative indicators for comprehensively assessing environmental, social, and economic sustainability. This study addresses this challenge by developing a set of indicators specifically tailored for quantitative assessments across all three dimensions of sustainability in the wheat and rice supply chains.

This study proposes an optimal set of indicators for assessing wheat and rice supply chain sustainability using the TBL framework. The literature review revealed numerous sustainability indicators available for both grains, with variations observed primarily in environmental dimensions, while similarities were noted in economic and social dimensions. These differences were attributed to the distinct production processes associated with wheat and rice.

The main objective was to identify a common set of indicators for social and economic dimensions across both grains while employing different indicators for environmental dimensions. The aim was to select a minimal yet effective set of indicators that offer comprehensive sustainability insights, are easily interpretable, and have readily available data. The systematic literature review identified 12 environmental indicators for the rice supply chain and 11 for the wheat supply chain, along with 10 indicators for social and economic dimensions.

An expert survey was conducted to refine the indicator set further, considering geographical variations. This survey aimed to optimise the number of indicators for efficient data management. The analysis of survey data resulted in the final set of indicators to be used in blockchain-based sustainability information management and reporting systems.

However, deriving the optimal set of indicators faced challenges due to dynamic variables such as soil quality, weather patterns, and socio-economic conditions specific to each region. Nonetheless, some indicators were consistently utilised in both academic literature and industry sustainability reports, providing valuable insights into environmental sustainability. These indicators were categorised into input indicators (e.g., fertilisers, pesticides, water, energy, machinery usage) and output indicators (e.g., greenhouse gas emissions, acidification, eutrophication).

While output indicators offer a deeper understanding, they require complex analysis processes, whereas input indicators are easier to maintain and compare, making them more accessible to the general population. The selection process also considered previous studies; e.g., Roy et al., in [14], identified sustainability indicators in the rice supply chain within a TBL framework. While many indicators proposed in this study aligned with Roy et al. in [14], this study focused on quantifiable and readily available data, resulting in variations, especially in the social dimension.

## 7. Limitations and Future Work

The selected indicators will be integrated into a sustainability index, necessitating a concise yet effective set of indicators to represent the sustainability of grain supply chains. This study highlights the challenge of balancing comprehensiveness with practicality in indicator selection within the TBL framework.

This study utilised an expert opinion survey to select sustainability indicators driven by expert perspectives, which may not always align with consumer preferences. Therefore, it is essential to consider the consumer viewpoint in the indicator selection process, especially since sustainability information can influence consumer decision-making.

The sustainability index calculation employed a simple arithmetic average based on normalised sustainability indicator values. However, this approach may be misleading in cases where less sustainable impact indicators are weighted equally to higher-valued sustainability indicators. Future research could explore weighted averages based on expert opinion to address this issue, although the current study focused more on understanding the importance of individual indicators rather than determining rankings or weights.

Benchmarking sustainability indicators is crucial for designing a sustainability matrix, providing a reference for evaluation and quantification. However, challenges arise in defining benchmarking for wheat and rice supply chain sustainability due to the lack of an official international standard. While benchmarking typically involves comparing practices and results across entities, the global production of rice and wheat in diverse environments and economic conditions complicates the process. Although this study did not include benchmarking values within its scope, future research could define benchmarks and integrate them into the proposed system.

Future research will focus on explicitly mapping the identified sustainability indicators to specific UN SDGs. This mapping will help establish clear linkages between the indicators and global sustainability priorities, providing a stronger policy and implementation context. By aligning the indicators with relevant SDG targets, future work will enable more targeted sustainability strategies and facilitate benchmarking for policymakers, industry stakeholders, and researchers across different regions.

## 8. Conclusions

This paper extensively reviews the indicator selection criteria, development methods, validation, and evaluation strategies for agricultural sustainability assessment to achieve UN sustainability goals. The paper’s findings include the proposal of an optimal set of indicators of the rice and wheat supply chain under TBL. A two-step approach was followed: a systematic literature review followed by expert opinion to optimise the indicator findings.

A plethora of indicators were employed in assessing the sustainability of wheat and rice supply chains, both within industry practices and scholarly literature. It is evident that the selection of sustainability indicators varies between grains, particularly concerning environmental sustainability aspects. As a result, utilising grain-specific environmental sustainability indicators offers greater reliability and deeper insights into sustainability concerns. Conversely, social and economic indicators exhibit similarities across wheat and rice supply chains, allowing for the adoption of a common set of indicators.

In conclusion, this study has introduced a comprehensive array of sustainability assessment indicators covering economic, environmental, and social dimensions throughout the entire life cycle of rice products. Emphasis has been placed on food production, recognising its substantial influence on sustainability. Our framework and the associated evaluation methods strive to provide a systematic and coherent approach to sustainability assessment.

Given the increasing impact of information technology and the rising awareness among consumers about sustainability, there is a growing demand for an efficient sustainability indicator. This indicator would empower consumers to make informed choices when buying sustainably produced rice and wheat. Integrating the proposed set of indicators with information dissemination technologies like blockchain can effectively meet this demand. These indicators aim to prevent information overload and serve as educational tools, enlightening consumers about the sustainability aspects of their food products.

## Figures and Tables

**Table 1:** Survey analysis of environment indicators in the rice supply chain.

S. No.	Indicator	Min.	Max.	Mean	Std. Deviation	Variance
1	Fertiliser used (kg/ha)	1	4	1.72	0.834	0.696
2	Integrated weed management (%)	1	5	1.92	1.066	1.136
3	Cropping program (%)	1	5	2	1.161	1.347
4	Packaging material/agri film (kg/ha)	1	5	2.08	1.158	1.34
5	Pesticide used (kg/ha)	1	5	2.12	1.206	1.455
6	Fuel used (L/ha)	1	5	2.16	1.299	1.688
7	Water used (L/ha)	1	5	2.18	1.224	1.498
8	Organic fertiliser used (kg/ha)	1	5	2.18	1.207	1.457
9	Co-product handling/waste by-product handling (CO_2_ eq)	1	5	2.18	1.155	1.334
10	Field emission (CO_2_ eq)	1	5	2.22	1.404	1.971
11	Electricity used (mj/ha)	1	5	2.26	1.175	1.38
12	Machinery used (HP/ha)	1	5	2.32	1.377	1.896
13	Seed production (CO_2_ eq)	1	5	2.34	1.272	1.617
14	Lime used (kg/ha)	1	5	2.36	1.241	1.541
15	Fertiliser produced on-farm (kg)	1	5	2.6	1.309	1.714

**Table 2:** Survey analysis of environment indicators in the wheat supply chain.

S. No.	Indicator	Min.	Max.	Mean	Std. Deviation	Variance
1	Water used (L/ha)	1	5	2.06	1.15	1.323
2	Pesticide used (kg/ha)	1	5	2.12	1.239	1.536
3	Cropping program (%)	1	5	2.14	1.088	1.184
4	Lime used (kg/ha)	1	5	2.16	1.149	1.321
5	Soil organic carbon loss (%)	1	5	2.18	1.207	1.457
6	Fertiliser used (kg/ha)	1	5	2.22	1.234	1.522
7	Integrated weed management (%)	1	5	2.22	1.298	1.685
8	Fuel used (L/ha)	1	5	2.24	1.222	1.492
9	Co-product handling/waste	1	5	2.24	1.135	1.288
10	Seed production (CO_2_ eq)	1	5	2.34	1.239	1.535
11	Electricity used (mj/ha)	1	5	2.4	1.178	1.388
12	Machinery used (HP/ha)	1	5	2.42	1.311	1.718
13	Fertiliser produced on-farm (kg)	1	5	2.62	1.26	1.587

**Table 3:** Survey analysis of economic indicators.

S. No.	Indicator	Max.	Min.	Mean	Std. Deviation	Variance
1	Revenue (AUD)	1	5	1.86	1.088	1.184
2	Land productivity (kg/ha)	1	5	1.94	1.15	1.323
3	Benefit-to-cost ratio (%)	1	5	2.04	0.903	0.815
4	Marketing opportunity (%)	1	5	2.06	0.978	0.956
5	Labour productivity (kg/labour)	1	5	2.14	1.03	1.062
6	Import dependency (%)	1	5	2.16	1.149	1.321
7	Profitability (AUD)	1	5	2.18	1.273	1.62
8	Revenue per family worker (AUD)	1	5	2.24	1.271	1.615
9	Use of chemical fertiliser (AUD/ha/year)	1	5	2.26	1.139	1.298
10	Production cost (AUD)	1	5	2.32	1.168	1.365

**Table 4:** Survey analysis of social indicators.

S. No.	Indicator	Min.	Max.	Mean	Std. Deviation	Variance
1	Accident rate (%)	1	5	2	1.088	1.184
2	Employment (persons/kg)	1	5	2.14	1.143	1.307
3	Training (binary)	1	5	2.14	1.01	1.021
4	Average wages (AUD)	1	5	2.18	1.137	1.293
5	Health(AUD/employee)	1	5	2.2	1.125	1.265
6	Community engagement	1	5	2.26	1.065	1.135
7	Education (level)	1	5	2.3	1.147	1.316
8	Gender equality (%)	1	5	2.32	1.22	1.487
9	Service to society (AUD)	1	5	2.6	1.212	1.469

## Data Availability

The original contributions presented in the study are included in the article, further inquiries can be directed to the corresponding author.

## Appendix A

	**Indicators**	**Unit**	**Definition**	**Counts**
**Environment**	Fertiliser used/type	kg/ha	Use of fertilisers in agriculture	23
	Fuel used (diesel)	L/ha	Total fossil fuels used in farming/transport	22
	Field emission	CO_2_ eq	Number of days crop was in a flooded condition	17
	Seed production	CO_2_ eq	Total emission from the production of seed	15
	Pesticide used	kg/ha	Use of pesticides in agriculture	14
	Electricity	mj/ha	Direct on-farm energy consumption/in processing	13
	Fertiliser production	kg/ha	Fertilised produced at the farm	9
	Machinery production	HP	Number of machines used and their ratings	8
	Water used	L/ha	Irrigation water application rate	8
	Organic fertiliser	kg/ha	Organic fertiliser used	8
	Co-product handling	CO_2_	How co-product was utilised	7
	Packaging material/agri film	kg/ha	The total packaging material used per hectare of farm/ product	6
**Economic**	Land productivity	kg/ha	Gross output per hectare	12
	Profitability	AUD or %	Market-based gross margin (fewer subsidies) per hectare	7
	Labour productivity	kg/labour	Income obtained per work unit of labour	6
	Income per family worker	$	Income per family member working on the farm	5
	Revenue	$	Total revenue of farm	4
	Production cost	$	Total input cost	4
	Import dependency	%	Ratio of import products to domestic products	4
	Viability	%	The farm is economically viable	3
	Use of chemical fertiliser	kg/ha	Crop yield (kg) per kg of fertiliser applied	3
	Marketing network	Likert scale	Availability of market for produced	3
**Social**	Knowledge/education	%	Average education level of farmers	12
	Employment	Number	Number of employees	9
	Average wages	AUD	Worker’s wage as compared to the national legal minimum wage	7
	Equality	%	Male female employee ratio	7
	Accident rate	%	Number of safety incidents	7
	Health	Likert scale	Working conditions of workers in agriculture and along the value chain	6
	Community engagement		Number of community engagement event	6
	Training		Training provided to a worker	5
	Social capital	Likert scale	The donation, trust, local, SCR	4
	Resources availability	Likert scale	Availability of resources to farm	3
